# Laparoscopic Adrenalectomy for the Treatment of Isolated Large Adrenal Gland Metastases from Malignant Melanoma: A Case Report

**DOI:** 10.7759/cureus.6020

**Published:** 2019-10-29

**Authors:** Bahadır Öz, Mustafa Gök, Erdoğan Sözüer, Uğur Topal, Figen Öztürk

**Affiliations:** 1 General Surgery, Erciyes University Faculty of Medicine, Kayseri, TUR; 2 Pathology, Erciyes University Faculty of Medicine, Kayseri, TUR

**Keywords:** laparoscopic adrenalectomy, isolated metastases, malignant melanoma, lateral approach

## Abstract

The adrenal gland is a common site for metastatic disease. However, isolated adrenal metastases (AM) are rare. We present a case of a rapidly enlarging adrenal mass with solitary distant metastasis from primary malignant melanoma. To our knowledge, the present case is the largest solitary mass that involves the adrenal gland from malignant melanoma in the literature. The present patient with a large adrenal mass (14 cm) was successfully operated by a transperitoneal laparoscopic approach. The surgeon's laparoscopic experience is more important than the lesion size for laparoscopic indications in selected patients.

## Introduction

The adrenal gland is a common site for metastatic disease. However, isolated adrenal metastases (AM) are rare [1]. The most common primary sites for adrenal metastases are renal cell carcinoma, malignant melanoma, and carcinoma of the lung and colon [2].

The advantages of laparoscopic adrenalectomy over traditional open surgery include less analgesic requirement, less blood loss, earlier recovery in terms of postoperative ileus, earlier resumption of a regular diet, shorter length of hospital stay, and earlier return to work [3]. The role of laparoscopic adrenalectomy for the excision of any malignant lesion remains controversial; however, it is generally believed that a solitary metastasis should be removed if morbidity is acceptable [4]. We have previously published our experience with laparoscopic adrenalectomy [5].

The objective of this study is to present our experience of laparoscopic adrenalectomy for isolated large adrenal metastasis from malignant melanoma.

## Case presentation

We present the case of a 45-year-old female with a previous history of cutaneous malignant melanoma of the left calf. She was diagnosed at five years earlier, had undergone lesion excision and superficial and deep lymph node dissection, and received radiation therapy at a dose of 300 cGy/day, for a total dose of 3000 cGy in 10 fractions. After two years, she received three doses of ipilimumab due to local recurrence in the inguinal region. Then, after four years, recurrences occurred in the left thigh and lymph nodes in the pelvic regions and abdominal anterior wall. She underwent excision and popliteal and pelvic lymph dissection.

**Figure 1:**
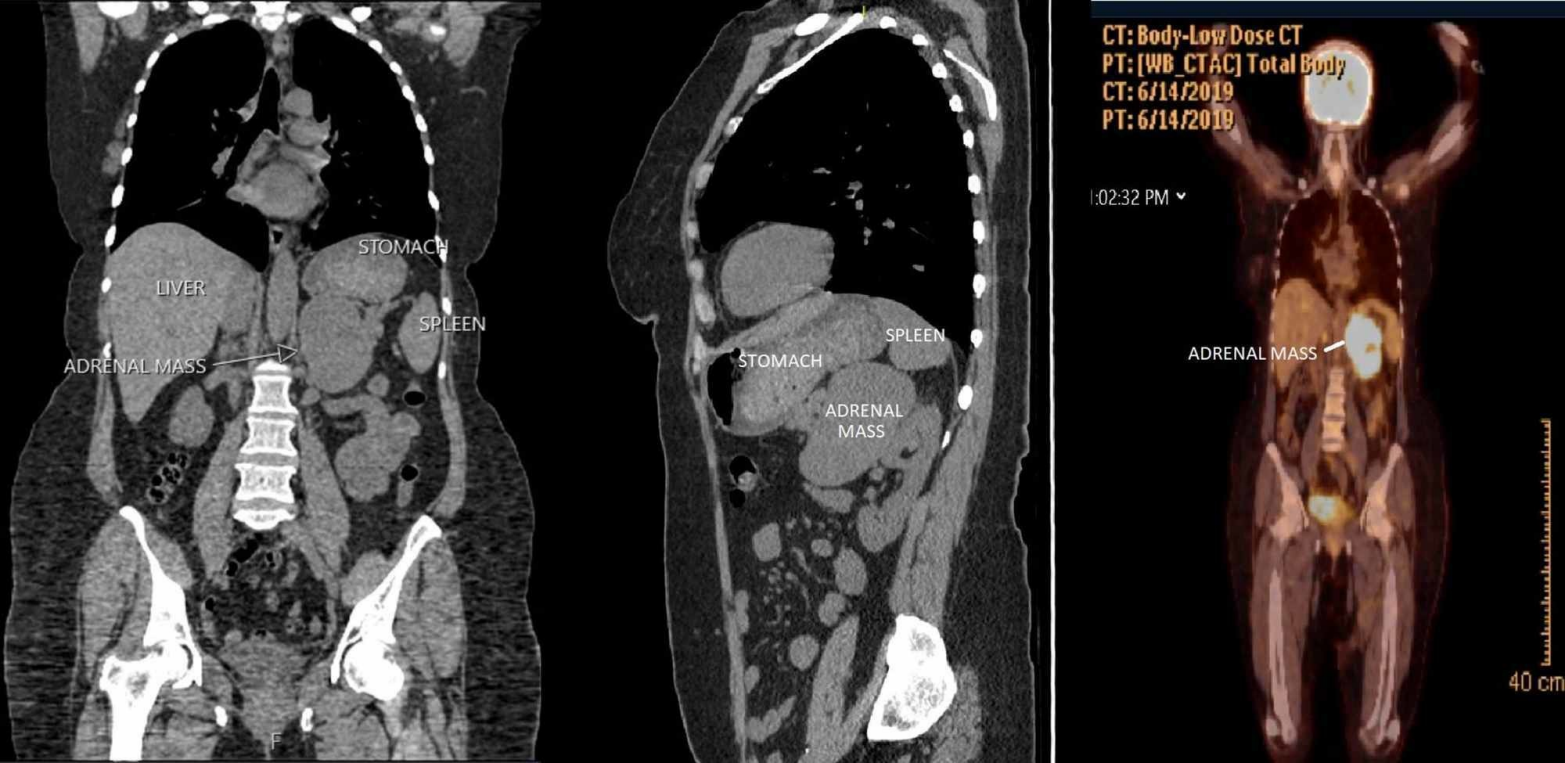
Abdominal computed tomography (CT) view with coronal (A) and sagittal section (B); positron emission tomography-CT (PET-CT) view with coronal section (C)

She had a history of epigastric quadrant pain and bloating, especially after meals. On physical examination, no peritoneal irritation was noted. Similarly, abdominal computed tomography (CT) (Figures 1A-1B) and positron emission tomography (PET)-CT showed a 6*7*12 cm mass with necrotic areas filling the adrenal and gastrosplenic area standardized uptake value (SUV) max value was 17.6 (intense metabolic activity) (Figure 1C). No abnormal fluorodeoxyglucose (FDG) uptake was observed in other areas of the body including lymph nodes or distant. There was no local invasion of adjacent organs. Complete hormonal tests were performed before surgery in patients with incidentally detected adrenal masses. Hormone active pathology was not detected under the supervision of the endocrinologist. A 24-hour urinary-free cortisol, 24-hour urinary metanephrines, normetanephrine, and vanillylmandelic acid levels and serum aldosterone-to-renin ratio levels were evaluated. These biochemical tests ruled out any endocrine dysfunction.

Considering the patient's history and imaging methods, a needle biopsy was not necessary and not suitable for definitive diagnosis due to the high suspicion of metastasis. The patient was prepared for the surgery and was operated on. During the operation, it was decided that the mass could be removed by a laparoscopic procedure.

**Figure 2:**
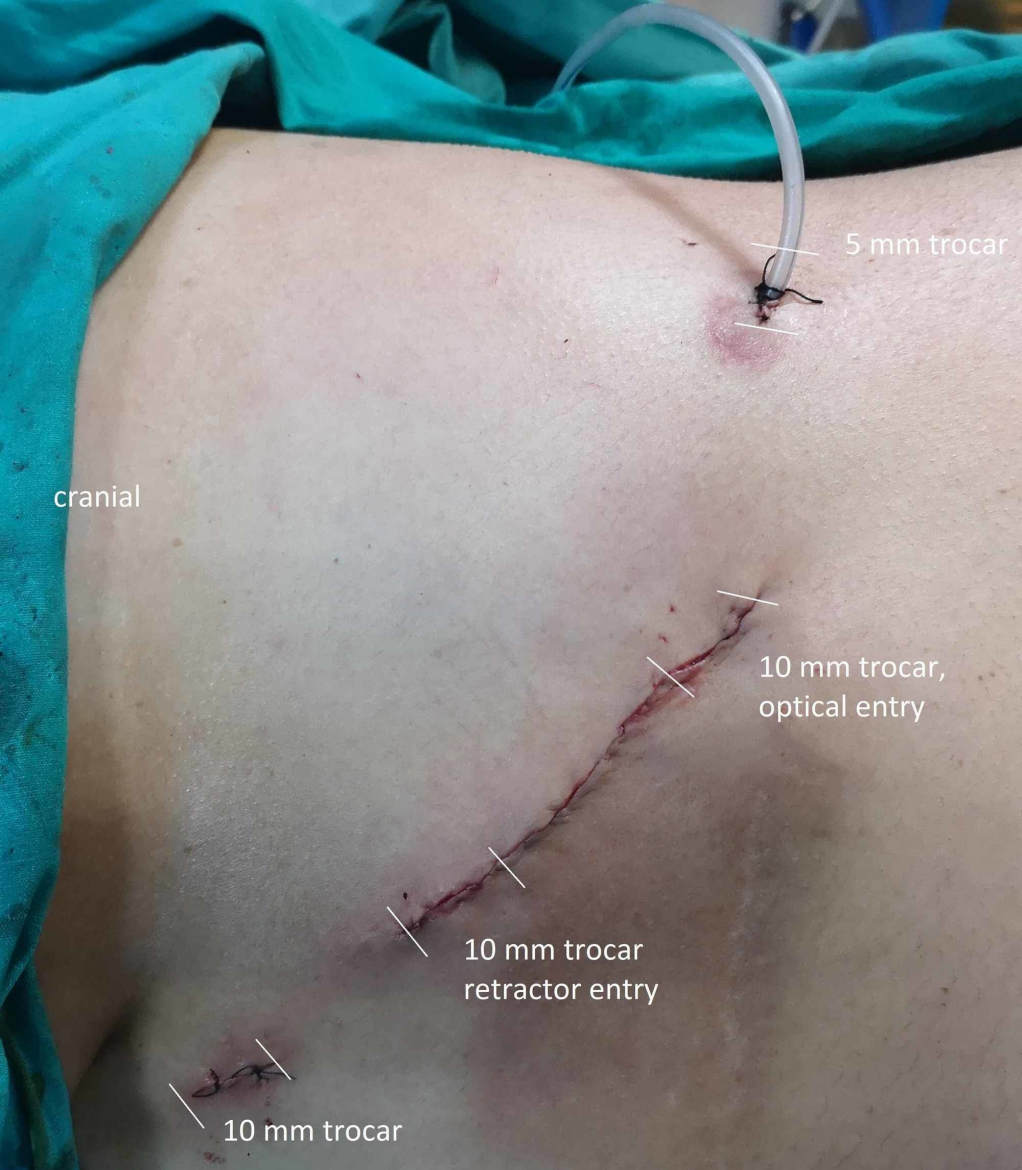
Placement of trocars

**Figure 3:**
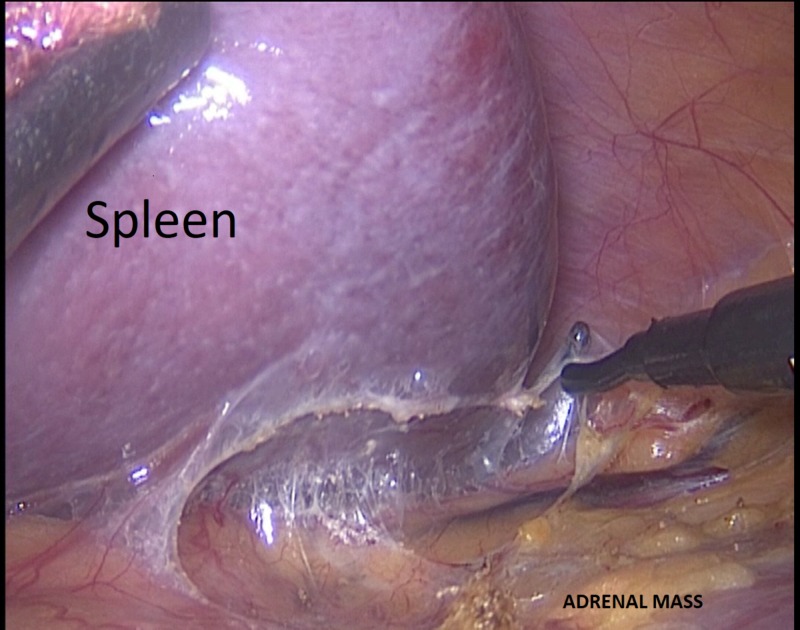
The mobilization of splenophrenic ligament

**Figure 4:**
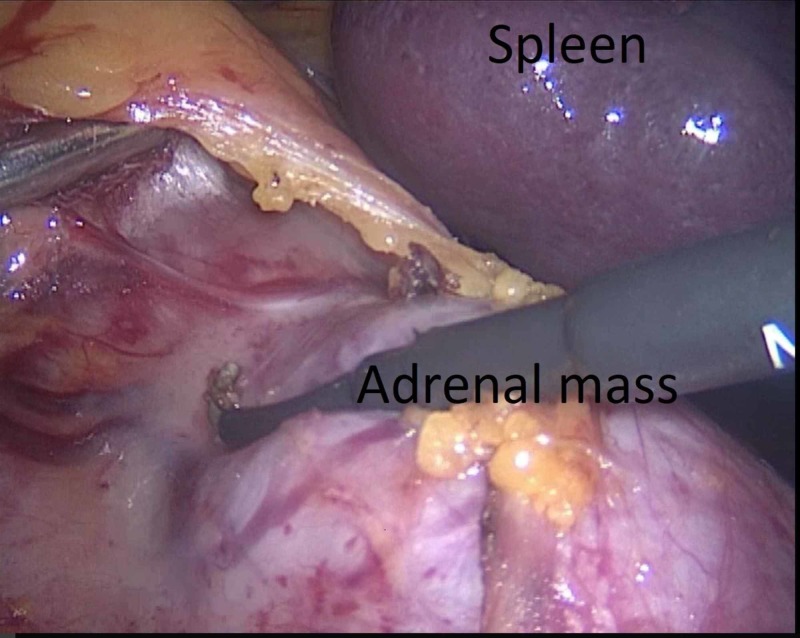
The mobilization of splenorenal ligament

**Figure 5:**
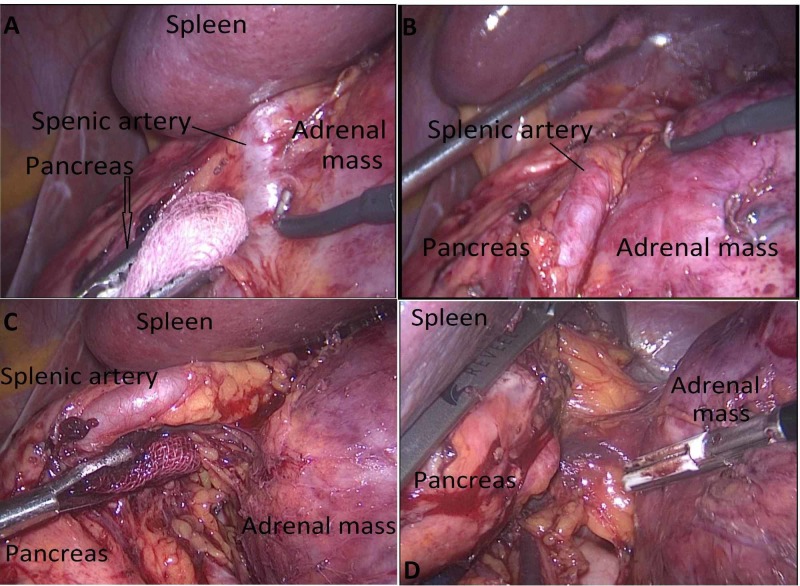
The lesion localization in the posterior of the tail of the pancreas and gastrocolic area (A), separation of the adrenal lesion from pancreatic tissue and splenic artery and vein (B,C), replacement of retractor (D)

**Figure 6:**
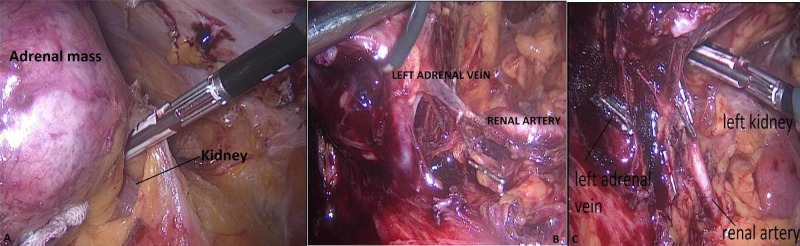
The closure of the gerota fascia (A), identification of left renal artery (B), ligation of the left adrenal vein (C)

**Figure 7:**
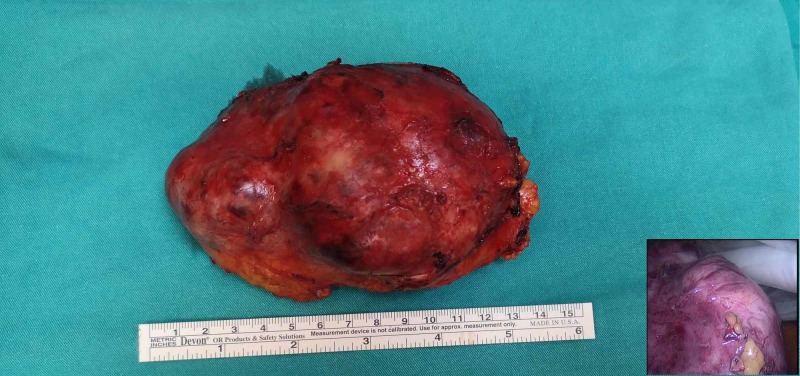
Specimen and the using of endobag (lower right corner)

The procedure was performed through a lateral transperitoneal laparoscopic approach using four ports. The patient was placed in the right lateral decubitus position. A Veress needle was inserted 3 cm under the costal margin at the anterior axillary line. Then, the pneumoperitoneum was created by the insufflation of carbon dioxide up to 14 mmHg. The 10-mm trocar was replaced by a 30° 10-mm camera. With 10-cm intervals, a second 5-mm and third 10-mm trocar were replaced on the midclavicular line and posterior axillary line, respectively, which are shown in Figure 2. The procedure was started with a superior lateral dissection. The splenophrenic, splenocolic, and splenorenal ligaments are identified and divided (Figures 3-4) In the meantime, a significant portion of the lesion was located in the posterior of the tail of the pancreas and gastrocolic area (Figure 5A). With careful dissection, the lesion was separated from the pancreatic tissue and splenic artery and vein (Figures 5B-5C). This did not allow for medial rotation of the spleen and tail of the pancreas, therefore, the fourth trocar was replaced between that of anterior axillary line and midclavicular line (Figure 5D). Then afterward, when the most medial was reached, the Gerota fascia was opened to identify the superior margin of the left renal vein (Figure 6A). This was followed medially until its junction with the inferior adrenal artery and vein was identified (Figure 6B). The adrenal vein was clipped at the level of the renal vein and divided (Figure 6C). The dissection was then continued in a medial-to-lateral and an inferior-to-superior manner. The left renal artery was seen in the posterior inferior part of the lesion and preserved (Figure 6B). The specimen was removed using an endobag with a 6-cm incision to maintain its capsule integrity (Figure 7). The incision was made by combining the additional trocar entry site and camera entry site (Figure 3). The adrenal lesion size was measured as 140 mm*70 mm (Figure 7). During dissection, the Hook and LigaSure instrument (Medtronic PLC, Minnesota, US) was generally used as a hybrid. The hemovac drain was placed in the operation area. Perioperative hypotension and a need for blood transfusion did not occur. The operative time was 142 minutes.

**Figure 8:**
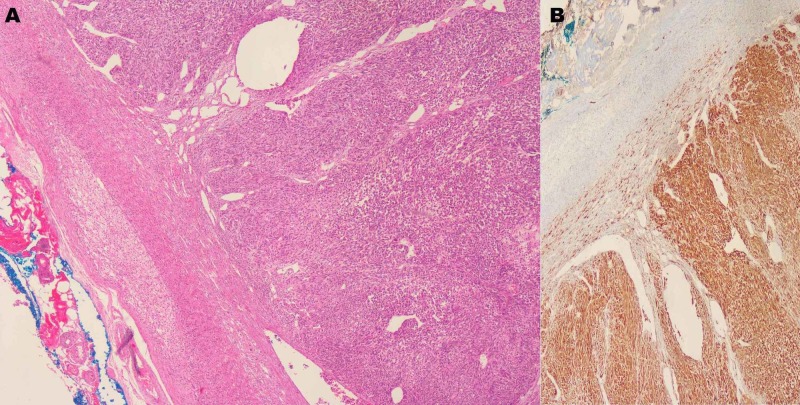
H&E (x40) (A) and positive staining for S100 (B) H&E: hematoxylin and eosin

Histopathology of the specimen reported malignant melanoma metastases to the adrenal gland. Immunohistochemical staining showed positive staining for S100, MITF, vimentin (pale), and HMB 45 (focal) and negative staining for cytokeratins (CK) (Figure 8).

Postoperatively, the patient was started on a liquid diet on the same day and a semisolid diet on the first day and was discharged with healing on the third postoperative day. No adjuvant treatment was recommended by Medical and Radiation Oncology.

## Discussion

Adrenal metastases commonly appear in patients with melanoma in the breast, lung, or gastrointestinal tract. Rarely, this may occur as isolated metastases to the adrenal gland [6].

We presented a case of a rapidly enlarging adrenal mass with solitary distant metastasis from a primary malignant melanoma using the laparoscopic approach. The role of laparoscopic adrenalectomy is still unclear for large and potentially malignant tumors [7]. Nowadays, laparoscopic surgery has become a good alternative to open surgery in patients with isolated metastases [8]. It is comparable to open surgery but with better postoperative comfort. It should be considered for the intention of complete resection [9].

When considering laparoscopic adrenalectomy for adrenal metastasis, oncological criteria should be maintained, such as early ligation of the main venous vessel, minimal instrumental manipulation for preserving the integrity of the capsule to reduce the risk of tumor cells' dissemination in the peritoneal cavity, and specimen removal using an endobag, to eliminate the possibility of the risk of trocar site contamination by tumor cells [10].

In the present study, the adrenal mass was removed with minimal manipulation using an endobag and a 6-cm laparotomy incision by combining a 2 trocar distance, preserving the integrity of the capsule. We also implemented the fourth trocar along with a retractor for the retraction of the spleen and pancreas, which allowed significant contribution to medial dissection during the laparoscopy.

There are few absolute contraindications for laparoscopic adrenalectomy but well-encapsulated adrenal masses without evidence of local invasion can be removed laparoscopically [11]. There was no local invasion or lymph node or distant metastases, which was confirmed by using contrast-enhanced CT and PET-CT in our case. This encouraged us to use the laparoscopic approach.

The lateral transabdominal approach offers the best visualization of major vessels adjacent to the adrenals. Lesion sizes of 12 cm to 14 cm have been reported as the upper limit for laparoscopic adrenalectomy in most of the studies [12]. Some surgeons have laparoscopically resected adrenal tumors up to 15 cm in size [13]. The present patient with a large adrenal mass (14 cm) was successfully operated using a transperitoneal laparoscopic approach.

## Conclusions

Isolated adrenal metastases are rarely found and should be carefully screened regarding distant or regional lymph nodes. It should also be evaluated whether the adrenal lesions involved are adjacent to organs. The surgeon's laparoscopic experience is more important rather than the lesion size for laparoscopic indications in selected patients. We think that a retractor applied from an additional trocar will be advantageous for the retraction of the spleen and pancreas in a large tumor, which would facilitate dissection toward the medial and caudal border.

## References

[1] Lam KY, Lo CY (2002). Metastatic tumours of the adrenal glands: a 30-year experience in a teaching hospital. Clin Endocrinol (Oxf).

[2] Bradley CT, Strong VE (2001). Surgical management of adrenal metastases. J Surg Oncol.

[3] Brunt LM, Doherty GM, Norton JA, Soper NJ, Quasebarth MA, Moley JF (1996). Laparoscopic adrenalectomy compared to open adrenalectomy for benign adrenal neoplasms. J Am Coll Surg.

[4] Sturgeon C, Leong SP, Duh QY (2004). Laparoscopic surgery for melanoma metastases to the adrenal gland. Expert Rev Anticancer Ther.

[5] Öz B, Akcan A, Emek E (2016). Laparoscopic surgery in functional and nonfunctional adrenal tumors: a single-center experience. Asian J Surg.

[6] Paul CA, Virgo KS, Wade TP, Audisio RA, Johnson FE (2000). Adrenalectomy for isolated adrenal metastases from non-adrenal cancer. Int J Oncol.

[7] Assalia A, Gagner M (2004). Laparoscopic adrenalectomy. Br J Surg.

[8] Pędziwiatr M, Wierdak M, Natkaniec M (2015). Laparoscopic transperitoneal lateral adrenalectomy for malignant and potentially malignant adrenal tumours. BMC Surg.

[9] Sebag F, Calzolari F, Harding J, Sierra M, Palazzo FF, Henry JF (2006). Isolated adrenal metastasis: the role of laparoscopic surgery. World J Surg.

[10] Feliciotti F, Paganini AM, Guerrieri M, Baldarelli M, De Sanctis A, Campagnacci R, Lezoche E (2003). Laparoscopic anterior adrenalectomy for the treatment of adrenal metastases. Surg Laparosc Endosc Percutan Tech.

[11] Brunt ML, Moley JF (2001). Adrenal incidentaloma. World J Surg.

[12] Zografos GN, Kothonidis K, Aggeli C (2007). Laparoscopic resection of large adrenal ganglioneuroma. JSLS.

[13] Kebebew E, Siperstein AE, Duh QY (2001). Laparoscopic adrenalectomy: the optimal surgical approach. J Laparoendosc Adv Surg Tech A.

